# Supplementation of dietary areca nut extract modulates the growth performance, cecal microbiota composition, and immune function in Wenchang chickens

**DOI:** 10.3389/fvets.2023.1278312

**Published:** 2023-12-12

**Authors:** Shiping Wang, Hong Wang, Qicheng Jiang, Jiahui Dai, Wenting Dai, Xiaoning Kang, Tieshan Xu, Xinli Zheng, An Fu, Zengyang Xing, Yiyong Chen, Zhongchun He, Lizhi Lu, Lihong Gu

**Affiliations:** ^1^Haikou Key Laboratory of Areca Processing Research, Institute of Agro-Products Processing and Design, Hainan Academy of Agricultural Sciences, Haikou, China; ^2^Institute of Animal Science and Veterinary Medicine, Hainan Academy of Agricultural Sciences, Haikou, China; ^3^Tropical Crops Genetic Resources Institute, Chinese Academy of Tropical Agricultural Sciences, Haikou, China; ^4^Wenchang City Wenchang Chicken Research Institute, Wenchang, China; ^5^Wenchang Spring of Dragon Wenchang Chicken Industrial Co., Ltd., Wenchang, China; ^6^Hainan Inheriting Good Taste Wenchang Chicken Industry Co., Ltd., Wenchang, China; ^7^Institute of Animal Husbandry and Veterinary Science, Zhejiang Academy of Agricultural Sciences, Hangzhou, China

**Keywords:** Wenchang chickens, *Areca catechu*, growth performance, metabonomics, cecal microbiota, MHC-BF1, Daxx upon analysis

## Abstract

**Introduction:**

The study was aimed at evaluating the effects of areca nut extract (ANE) on the growth performance, cecal microbiota, and immunity of Wenchang chickens.

**Methods:**

For this study, 42-day-old healthy Wenchang chickens (*n* = 450) with similar body weight were chosen. The animals were randomly divided into five groups, with six replicates per group and 15 chickens per replicate. One group was fed a basal diet (control; CCK). The remaining four groups were fed a basal diet supplemented with varying ANE concentrations: 0.038, 0.063, 0.100, and 0.151 g/kg, with the groups denoted as CNT1, CNT2, CNT3, and CNT4, respectively. The feeding experiment lasted 35 days. The ligated cecum segments of the control and experimental groups were collected for metabolomic and metagenomic analysis, while the bone marrow samples were extracted for tandem mass tag (TMT)-based proteomic analysis.

**Results:**

All the experimental groups exhibited significantly higher average daily gain (ADG) and significantly lower feed-to-weight (F/G) ratios than CCK. Metabolomic screening of the cecum contents revealed the presence of 544 differential metabolites, including several gut health–related metabolites, such as xanthine, hydroxy hypoxanthine, 2,5-dimethylhydrazine, ganoderic acid, and 2-aminohexanoic acid. Metagenomic analysis of the cecum contents showed an upregulation in the abundance of *Prevotella* spp. in the experimental groups. However, we observed no significant differences in the abundances of other cecal microbes at phylum and genus levels. Furthermore, we observed significant associations between *Prevotella* spp. and the differentially abundant metabolites, such as cherubins, thiaburimamide, and 3,4-dihydroxy-L-phenylalanine, (r)-mevalonate, 5-O-methylalloptaeroxylin, nalidixic acid, and deoxyloganin (*p* < 0.05). Proteomic analysis revealed that the differentially expressed proteins (such as interferon-induced protein with tetratricopeptide repeats 5 (IFIT5), MHC-BF1, and death domain-associated protein (Daxx)) in the bone marrow of the chickens were primarily enriched in the immune network for IgA production and B cell receptor signaling pathway.

**Conclusion:**

In conclusion, dietary ANE supplementation was found to enhance metabolic activity and energy utilization, improve growth performance, modulate cecal microbiota, and strengthen the immunity of Wenchang chickens.

## 1 Introduction

Areca nut is a tropical crop widely distributed across southeast Asia, including China, India, and Malaysia (1). It is extensively used as a component of traditional Chinese medicine (2). The active compounds in areca nut include arecoline and polyphenols (3). This crop has been shown to exhibit anti-inflammatory, analgesic, wound-healing, anti-HIV, antioxidant, and antimicrobial properties (4–7). In addition, the primary component, arecoline, is responsible for the anti-parasitic, anti-depressive, and anti-fatigue properties of areca nut (8, 9). Furthermore, areca nut consumption can also enhance gastrointestinal motility (10–14).

Wenchang chicken is an important breed of meat chicken known for its excellent meat quality, high intramuscular fat, and moderate subcutaneous fat (15, 16). Improvements in growth performance, gut health, and immunity are crucial for Wenchang chicken production. Previous studies have shown that the conventional practice of supplementing poultry feed with antibiotics to promote weight gain has led to the development of bacterial antibiotic resistance (17). Hence, many countries, including China, have prohibited the utilization of antibiotics as growth promoters for food animals, triggering research for exploring alternatives to such antibiotics (18). Currently, several dietary supplements, including enzyme preparations, amino acids, probiotics, prebiotics, and botanical natural extracts, are being investigated (19, 20). These supplements reportedly exhibit beneficial effects in certain aspects. For instance, dietary supplementation of 0.05% putrescine has been shown to enhance the growth performance and meat quality of Wenchang chickens (21). In addition, the direct supplementation of probiotics, such as lactic acid bacteria, bifidobacteria, and saccharomyces, has been shown to substantially enhance the gut functionality and growth performance of chickens (22). Some studies have explored the anti-parasitic effects of areca nut on chicken (23, 24). However, to the best of our knowledge, the effects of areca nut on the growth performance, cecal microbiota, and immune function of Wenchang chicken have not yet been investigated, which impedes the use of areca nut in Wenchang chicken raising. In the current study, we evaluated the effects of areca nut extract (ANE) on the growth performance, cecal microbiota, and immune function of Wenchang chickens. Our study could be used as a reference to explore the effects of using areca nuts as a dietary supplement for raising Wenchang chickens.

## 2 Materials and methods

### 2.1 Animal ethics

All procedures were conducted in accordance with the Regulations for the Administration of Affairs Concerning Experimental Animals (Ministry of Science and Technology, China, revised in March 2017). The protocol was approved by the Institutional Animal Care and Use Committee at the Experimental Animal Center of Hainan Academy of Agricultural Science (HNXMSY-20220633).

### 2.2 Extract preparation

#### 2.2.1 Preparation of ANE

Areca nuts were provided by the Institute of Processing and Design of Agroproducts, Hainan Academy of Agricultural Sciences. The dried areca nuts were ground using a high-speed pulverizer. H_2_O was used as the extraction solvent with a material-to-liquid ratio of 1:20. The ground nut powder was submerged for 24 h at a temperature of 60°C. After extraction, the mixture was filtered using a 300-mesh sieve and then subjected to rotary evaporation at 60°C. Finally, the residue was dried in an oven until it formed a paste-like consistency.

#### 2.2.2 Quantification of arecoline and polyphenols in ANE

Arecoline was quantified using high-performance liquid chromatography (HPLC), as described previously, with some modifications (25). For HPLC, we used a Waters UPLC HSS T3 chromatographic column (100 mm × 2.1 mm × 1.8 μm), a 215-nm wavelength, a column temperature of 30°C, a flow rate of 0.15 mL/min, a sample volume of 10 μL, and a mobile phase comprising 0.1% phosphoric acid-acetonitrile in a 65:35 ratio. Furthermore, areca nut polyphenols were detected using enzyme-linked immunosorbent assay (ELISA). The ELISA kit was purchased from Suzhou Michy Biology Technology Co., Ltd. The HPLC analysis and ELISA showed that the extracted ANE contained 8.75 μg/g and 8.86 mg/g of arecoline and phenol, respectively. The secondary metabolites in ANE were detected using liquid chromatography-mass spectrometry (LC–MS) (Supplementary Table S1).

#### 2.2.3 Chicken feed administration

We used 450 healthy 42-day-old Wenchang chickens with consistent genetic backgrounds and similar body weight. The chickens were randomly allocated into five groups. Each group comprised six replicates, with 15 chickens in each replicate. One group was fed with basal diet (control group; CCK). The remaining four groups were fed with basal diet supplemented with 0.038, 0.063, 0.100, and 0.151 g ANE per kg basal diet, with the groups denoted as CNT1, CNT2, CNT3, and CNT4, respectively. The feeding experiment lasted 35 days. The chickens had free access to feed and water, received routine immunizations, and were managed per standard feeding practices. The body weights of the groups were measured on the first and 35th day of the experiment (BW1 and BW35, respectively). In addition, we measured the average daily gain (ADG), average daily feed intake (ADFI), and feed-to-weight (F/G) ratio for all groups. The composition of the basal diet and nutritional levels are shown in Table 1. For further analyses, we selected the experimental group with the lowest F/G ratio (which was CNT4) and CCK.

**Table 1:** Feed composition and nutritional levels of basal diet (dry material basis).

Feed ingredients	Content	Nutritional levels	Content
Corn (%)	62.20	Crude protein (%)	18.00
Soybean meal (%)	27.80	Metabolizable energy (MJ/kg)	14.43
Wheat (%)	3.75	Methionine (%)	0.41
Fish meal (%)	1.00	Lysine (%)	0.97
Soy oil (%)	2.25	Arginine (%)	1.05
Vitamin A (IU/kg)	9,000	Calcium (%)	0.76
Vitamin D (IU/kg)	500	Phosphorus (%)	0.55
Vitamin E (IU/kg)	35	Sodium (%)	0.16
Vitamin K (mg/kg)	2.2	Chlorine (%)	0.16

### 2.3 Extraction of cecum and bone marrow samples

Eight chickens were selected randomly from CCK and CNT4. Cecal tissue was carefully extracted From each selected chicken and immediately ligated using sterile sutures to ensure sufficient cecum content. The ligated cecum segment was then excised. Simultaneously, the bone marrow of each chicken was also removed and promptly transferred into sterile 2-mL cryogenic storage tubes. These tubes were then stored at −80°C for subsequent analysis. The cecal samples were subjected to metabolomic and metagenomic analyses, while the bone marrow samples were subjected to tandem mass tag (TMT)-proteomics.

### 2.4 Metabolomic analysis using LC–MS

One hundred milligrams of each cecal sample was thawed on ice and mixed with 1 mL of pre-cooled 50% methanol. The mixtures were then vortexed for 1 min, followed by incubation for 10 min at room temperature. The obtained extraction mixtures were stored at −20°C overnight. After centrifugation at 4,000 × g for 20 min, the supernatants were transferred to 96-well plates and stored at −80°C for LC–MS. LC–MS was performed using the Vanquish Flex UHPLC system (Thermo Fisher Scientific, Bremen, Germany) coupled with a high-resolution tandem mass spectrometer Q-Exactive (Thermo Scientific). For sample resolution, we used an ACQUITY UPLC T3 column and a column temperature of 35°C. In addition, we used the Q-Exactive mass spectrometer operated in both positive and negative ion modes, collecting precursor spectra from m/z 70 to 1,050 at a resolution of 70,000 with an automatic gain control (AGC) target of 3e6. Fragment spectra were collected at a resolution of 17,500 with an AGC target of 1e5. A QC sample was acquired after a run of every 10 samples to assess system stability. The XCMS software was used for peak picking, grouping, retention time correction, and annotation of isotopes and adducts. The MS data was converted into mzXML format and processed using XCMS, CAMERA, and the metaX toolbox in R software. The retention time (RT) and m/z data were combined for metabolite identification. The Kyoto Encyclopedia of Genes and Genomes (KEGG) and Human Metabolome Database (HMDB) were used for metabolite annotation, with a mass difference threshold of 10 ppm. We used t-tests to detect the differences in the measured concentrations with Benjamini-Hochberg FDR correction. Partial least squares-discriminant analysis (PLS-DA) with variable importance in projection (VIP) cutoff of 1.0 was used to discriminate the features between the groups.

### 2.5 Metagenomic analysis

Metagenomic DNA was extracted from various samples using the E.Z.N.A.^®^ Stool DNA kit. The extracted DNA was eluted in 50 μL of elution buffer and stored at −80°C until PCR analysis. DNA libraries were prepared with the TruSeq Nano DNA LT Library Preparation kit (FC-121-4001). The DNA was fragmented and processed for sequencing. Sequencing adapters were removed, low-quality reads were trimmed, and host genome contamination was eliminated. Quality-filtered reads were assembled *de novo* to construct the metagenome for each sample. Next, coding sequences (CDSs) were predicted from metagenomic contigs using MetaGeneMark v3.26. CDSs from all samples were clustered into unigenes using CD-HIT v4.6.1. Unigene abundance for each sample was estimated by calculating transcripts per million (TPM) based on the number of aligned reads using bowtie2 v2.2.0. The lowest common ancestor taxonomy and functional annotations for unigenes were obtained by aligning the unigenes against the National Center for Biotechnology Information (NCBI) Non-Redundant (NR) database using DIAMOND v0.9.14. Differential analysis was conducted at various levels using statistical tests based on taxonomic and functional annotations of unigenes and their abundance profiles.

### 2.6 TMT-labeled quantitative proteomic analysis

TMT labeling was used to analyze the proteomic profiles of the bone marrow samples. First, the bone marrow samples were weighed and transferred to centrifuge tubes. Then, steel balls, urea-containing lysis solution, Tris–HCl, and Roche cocktail were added to the tubes. The tubes were placed on ice for 5 min and then homogenized using a tissue laser. Next, the solutions were centrifuged, and the supernatant was collected. Then, dithiothreitol (DTT) was added to the samples. The solution was then placed in a water bath at 37°C for 1 h. Finally, iodoacetamide (IAA) was added to the samples. The solutions were then incubated for 30 min in the dark. The Bradford method was used for protein quantification. The protein samples were then separated via SDS-PAGE. Next, the TMTpro-16plex labels were dissolved in acetonitrile and added to each sample at a specific ratio for TMT labeling. The labeled peptides from each sample were mixed and diluted. The fractions were collected, freeze-dried, reconstituted in 0.1% formic acid, and centrifuged. The supernatant was collected and injected into an Agilent ZORBAX 300Extend-C18 column for separation. The separated peptides were ionized and transferred to a mass spectrometer for detection. The MaxQuant software was used for data analysis. The TMT-labeled mass spectrometry (MS)/MS raw data was analyzed, with specific settings, to measure enzyme digestion parameters, modifications, mass tolerances, and false discovery rate (FDR) thresholds. Protein sequences were searched from the Uniprot database, and contaminants or reverse sequences were removed. The R software was used for normalization, hierarchical clustering, principal component analysis (PCA), t-tests for differential analysis, and enrichment analysis for annotation.

### 2.7 Statistical analysis

The growth performance of Wenchang chickens was analyzed using IBM SPSS Statistics 27.0 (IBM, USA). The results were presented as mean ± standard deviation. One-way analysis of variance (ANOVA) was used for intergroup comparisons, with a significance level set at *p* < 0.05. Duncan’s multiple range test for multiple comparisons was used for post-hoc analysis.

## 3 Results

### 3.1 Effects of dietary ANE supplementation on Wenchang chicken growth

As shown in Table 2, no significant differences were observed in the BW1 of any of the groups. However, BW35 of CNT1 was significantly higher than that of CCK (*p* < 0.05). Furthermore, we observed significantly higher ADGs in all experimental groups compared to CCK (CNT1–CNT3 vs. CCK: *p* < 0.05 and CNT4 vs. CCK: *p* < 0.01). In addition, we observed significantly lower F/G ratios in all experimental groups compared to CCK (CNT1–CNT3 vs. CCK: *p* < 0.05 and CNT4 vs. CCK: *p* < 0.01).

**Table 2:** Effects of dietary ANE supplementation on the growth performance of Wenchang chickens.

Parameters	CCK	CNT1	CNT2	CNT3	CNT4	*p*
BW1 (g)	672.2 ± 16.54	661.1 ± 24.37	664.4 ± 25.51	667.8 ± 23.35	660.0 ± 15.18	0.782
BW35 (g)	1235.0 ± 35.07^abc^	1358.3 ± 38.69^a^	1296.7 ± 81.4^bc^	1296.7 ± 25.82^bc^	1330.0 ± 49.80^ab^	0.027
ADG (g/d)	18.75 ± 1.43 ^c^	22.57 ± 1.36^ab^	21.06 ± 2.00^b^	21.28 ± 1.32^b^	23.02 ± 1.88^a^	0.009
ADFI (g/d)	45.13 ± 2.00^b^	53.38 ± 2.56^ab^	47.24 ± 5.56^b^	46.36 ± 1.67^b^	48.24 ± 2.54^b^	0.011
F/G (g/g)	2.41 ± 0.18^a^	2.37 ± 0.14^a^	2.24 ± 0.23^b^	2.19 ± 0.21^b^	2.11 ± 0.23^c^	0.025

BW1, the body weight of the group on the first day of the feeding experiment; BW35, the body weight of the group on the 35th day of feeing experiment; ADG, average daily gain; ADFI, average daily feed intake; F/G, feed intake/body weight gain. CCK, control group; CNT1, group fed with basal diet + 0.038 g ANE per kg basal diet; CNT2, group fed with basal diet + 0.063 g ANE per kg basal diet; CNT3, group fed with basal diet + 0.100 g ANE per kg basal diet; CNT4, group fed with basal diet + 0.151 g ANE per kg basal diet. Lowercase letters in superscript indicate significant differences (*p* < 0.05).

### 3.2 Metabolomic analysis of cecal samples

#### 3.2.1 Overview of metabolomic sequencing

**Figure 1:**
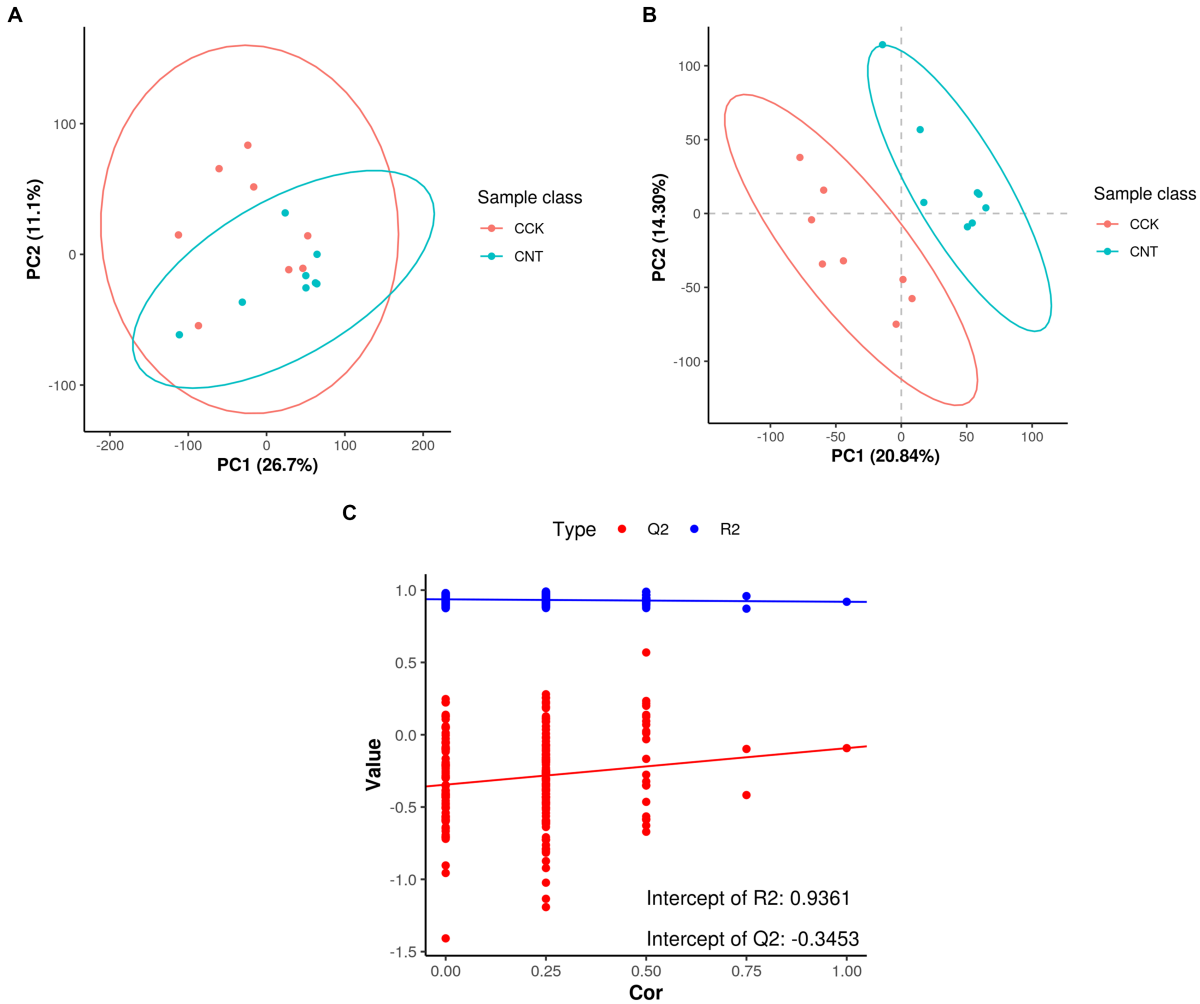
Score scatter plots of principal component analysis (PCA) and partial least squares discrimination analysis (PLS-DA) models for CCK and CNT4. **(A)** PCA between CCK and CNT4. **(B)** PLS-DA between CCK and CNT4. **(C)** Permutation test for PLS-DA.

The retention times and corresponding peak intensities in the chromatograms for the cecal samples of CCK and CNT4 were found to be reproducible (Supplementary Figure S1). Comparison of the mass spectra of the cecal samples with the library and reference compounds revealed a total of 24,538 metabolite ion peaks in all groups. Of these, 12,657 and 11,881 peaks were detected in the positive and negative scan modes, with 468 and 744 metabolite ions annotated to secondary metabolites, respectively (Supplementary Table S2). The detected metabolites were primarily classified into benzene and its derivatives, carboxylic acids and their derivatives, saturated hydrocarbons, pyrrolidine derivatives, lipids, and organic acids. PCA results showed that the quality control samples were clustered around the original samples (Figure 1A), indicating minimal instrument error during MS detection. The samples from CCK and CNT4 were mostly within a 95% confidence interval, indicating excellent repeatability and reliability of the results within each group. Furthermore, an orthogonal partial least squares-discriminant analysis (OPLS-DA) was constructed to achieve higher-level separation of the components and better understand the variables responsible for classification. OPLS-DA results showed separation between CCK and CNT4 (Figures 1B,C; R2Y = 0.9361 and Q2 = −0.3453), indicating significant differences between CCK and CNT4 at the metabolite level (Figure 1C).

#### 3.2.2 Identification and analysis of differential metabolites (DMs)

**Figure 2:**
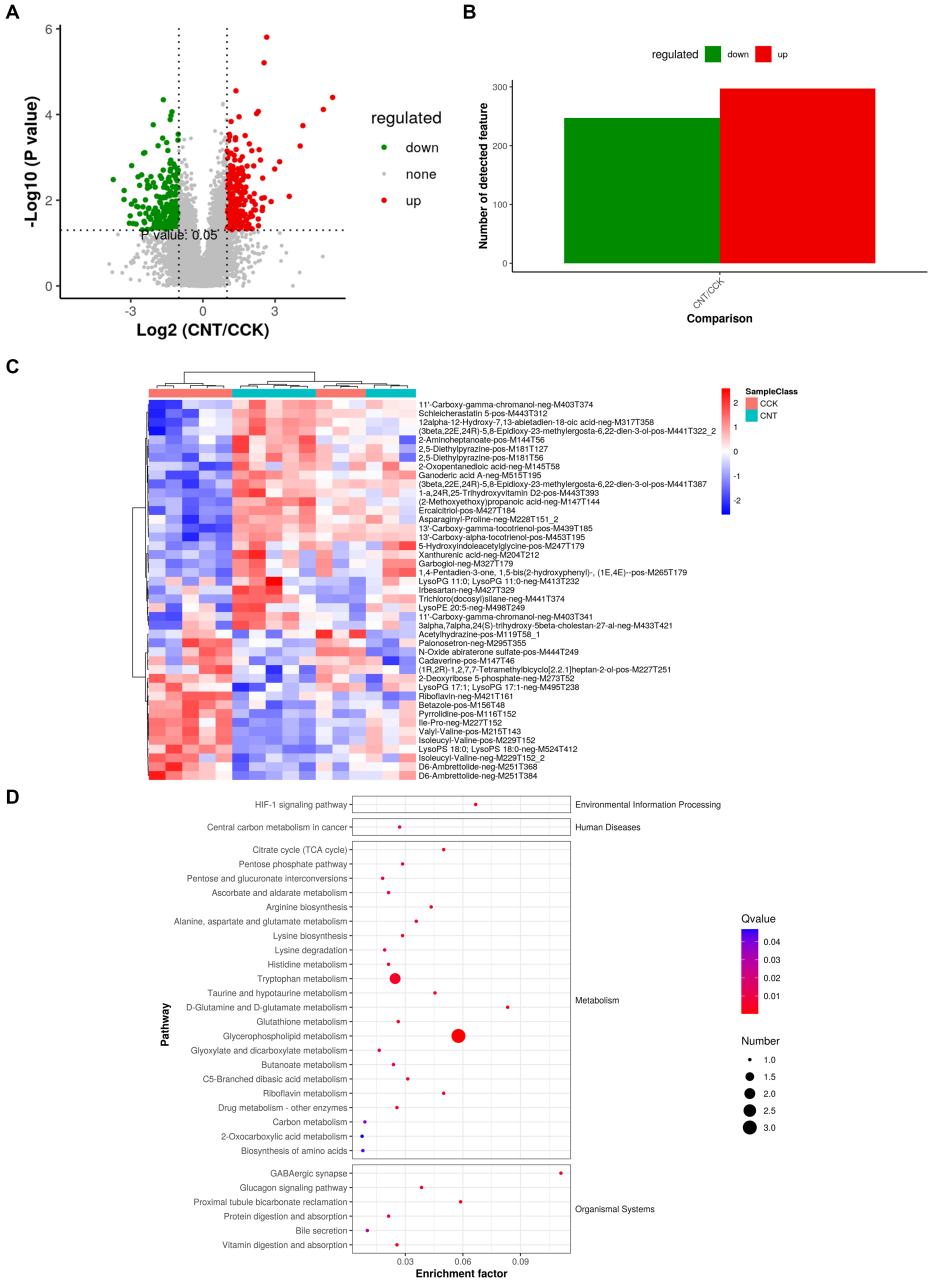
Multivariate analysis of untargeted metabolomics data. **(A)** Volcano plots for the model-separated metabolites with variable importance in projection (VIP) > 1 and *p* < 0.05. **(B)** The bar chart illustrates differential metabolite (DM) ion abundances. **(C)** Heatmap visualization of the DMs between CCK and CNT4. The color denotes the abundance of metabolites, with red and blue colors representing the highest and lowest abundances, respectively. **(D)** Scatter plots representing KEGG pathway enrichment for the DMs between CCK and CNT4.

Comparing the metabolites of CCK and CNT4 with the criteria of VIP > 1 and *p* < 0.05 revealed 544 DMs, with 297 upregulated and 247 downregulated metabolites in CNT4 (Figures 2A,B). Significantly modified metabolites after ANE feeding included xanthine, hydroxy hypoxanthine, 2,5-dimethylhydrazine, and pyrrolidine (Figure 2C). The selected DMs were subjected to pathway analysis, which revealed the involvement of these DMs in glycerophospholipid metabolism, D-glutamine metabolism, D-glutamate metabolism, and HIF-1 signaling pathways. Thus, ANE consumption was associated with the enhancement of metabolic pathways (Figure 2D).

### 3.3 Metagenomic analysis of cecal microbiota

**Figure 3:**
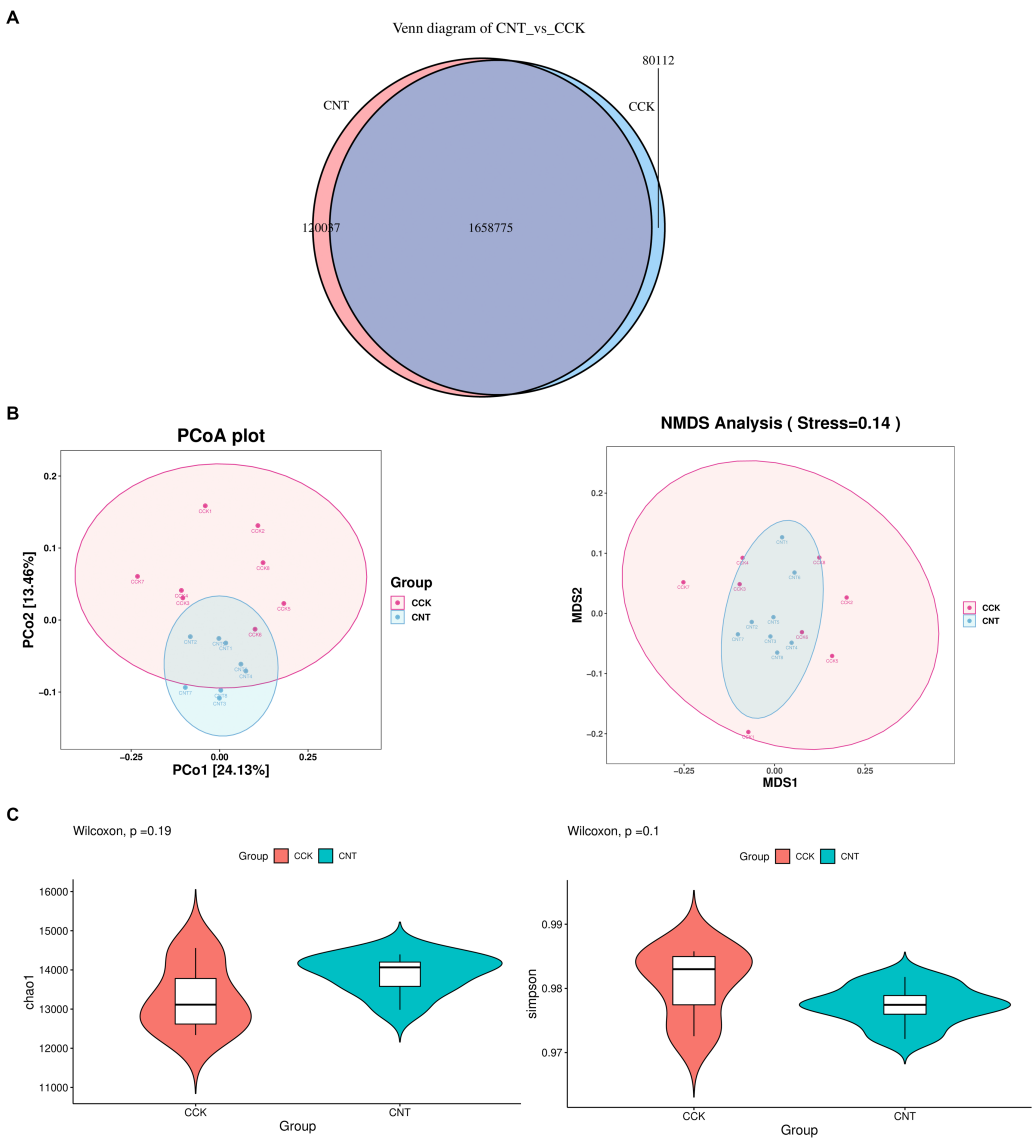
The differences in the gut microbiota of CCK and CNT4 based on the metagenomics data. **(A)** Venn diagram representing the number of genes identified in CCK and CNT4. **(B)** Principal coordinate analysis (PCoA) and non-metric multidimensional scaling (NMDS) of the microbiota based on the Bray-Curtis method. **(C)** The α-diversity indexes of the cecal microbiota in CCK and CNT4.

**Figure 4:**
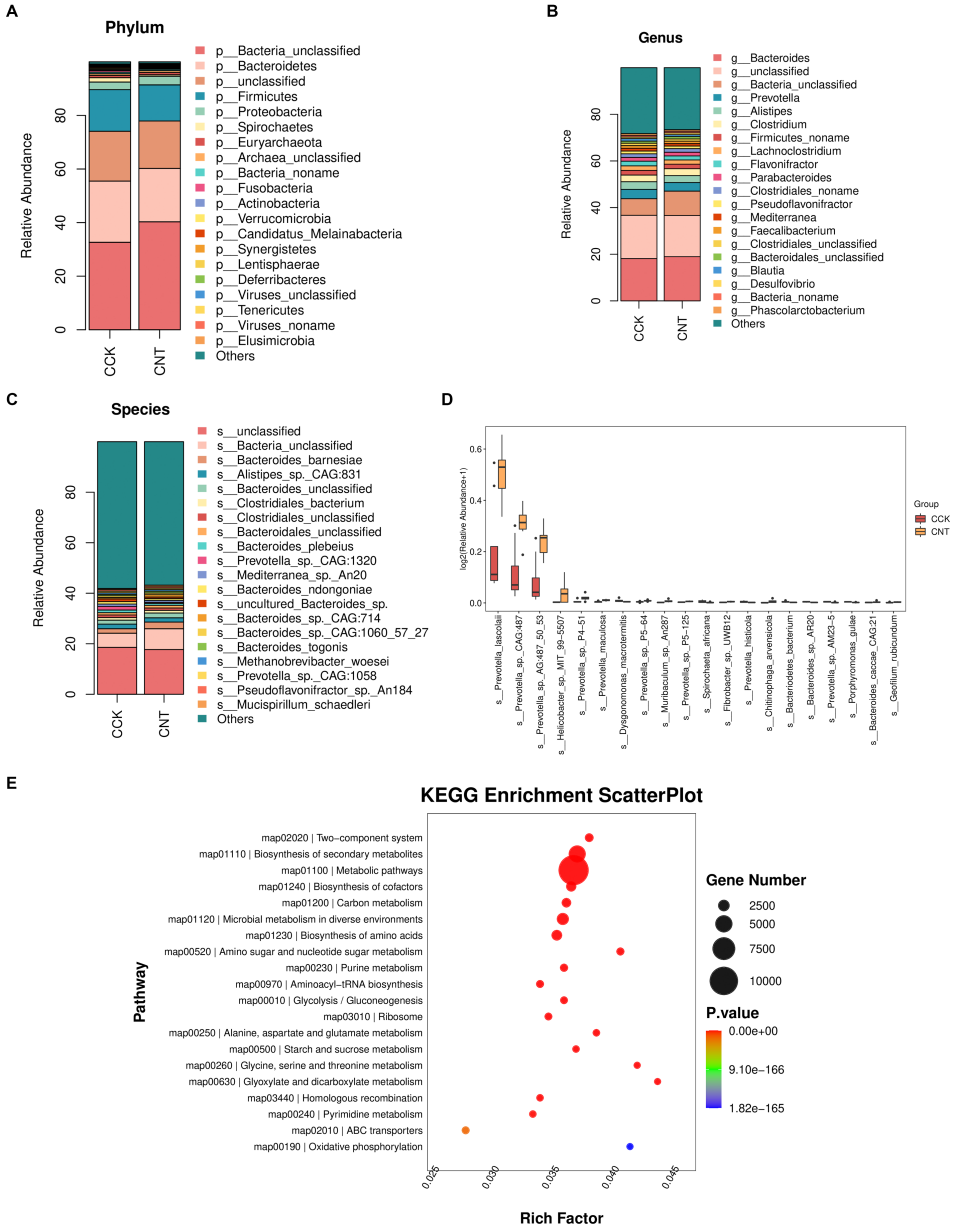
Relative abundance and functional enrichment of cecal microorganisms. The most abundant taxa of cecal microbiota at the **(A)** phylum, **(B)** genus, and **(C)** species levels. **(D)** The top 20 most abundant microbial species with significantly different abundances between CCK and CNT4. **(E)** Scatter plots representing the KEGG pathway enrichment of the differentially expressed unigenes between CCK and CNT.

The cecum contents of CCK and CNT were subjected to high-throughput sequencing. We obtained 84.4 Gb of clean metagenomic data after quality control and data filtering (Supplementary Table S3). For the Core-Pan genes, the sparse curve gradually flattened as the data approaches, indicating that the collected samples met the requirements for subsequent bioinformatic analysis. Gene-level analysis performed using a non-redundant gene set (Supplementary Figure S2) revealed a total of 1,858,924 genes in both groups. Venn diagram analysis indicated 80,112 and 120,037 unique genes in CCK and CNT, respectively (Figure 3A). Bray-Curtis-based principal coordinate analysis (PCoA) and non-metric multidimensional scaling (NMDS) were used to study the similarity in the microbial communities isolated from the cecal samples of both groups. Violin plots were generated based on Chao1 and Simpson indices showed no significant differences in microbial community composition and diversity between both groups. This finding suggested that ANE treatment might not impact the cecal microbiota in Wenchang chickens (Figures 3B,C). At the phylum level, the top 10 most abundant taxa in the cecal microbial community were Bacteroides, Firmicutes, Proteobacteria, Spirochaetes, Euryarchaeota, Fusobacteria, Actinobacteria, Verrucomicrobia, Candidatus Melainabacteria, and Synergistetes (Figure 4A). The CNT samples comprised relatively lower abundances of bacteroidetes and spirochetes compared to the CCK samples (*p* < 0.05). At the genus level, the top four most abundant taxa in the cecal microbial community were *Bacteroides*, *Prevotella*, *Alistipes*, and *Clostridium*, with no significant differences in their relative abundances between CCK and CNT4 (Figure 4B). At the species level, the top four most abundant taxa in the cecal microbial community included *Alistipes* sp. CAG:831, *Bacteroides barnesiae*, *Prevotella* sp. CAG:1320, and *Bacteroides plebeiu* (Figure 4C). Then, we selected the top 20 species with significantly different relative abundances and grouped them using box plots. Of these 20 species, *Prevotella* spp. was the most abundant in the cecal microbiota of ANE-fed Wenchang chickens (Figure 4D). The differentially expressed unigenes, retrieved from the KEGG database, were found to be involved in the biosynthesis and metabolism of secondary metabolites. They were primarily enriched in amino sugar and nucleotide sugar metabolism; glycine, serine, and threonine metabolism; and glyoxylate and dicarboxylate metabolism (Figure 4E).

### 3.4 Correlation between cecal microbiota and host metabolism

**Figure 5:**
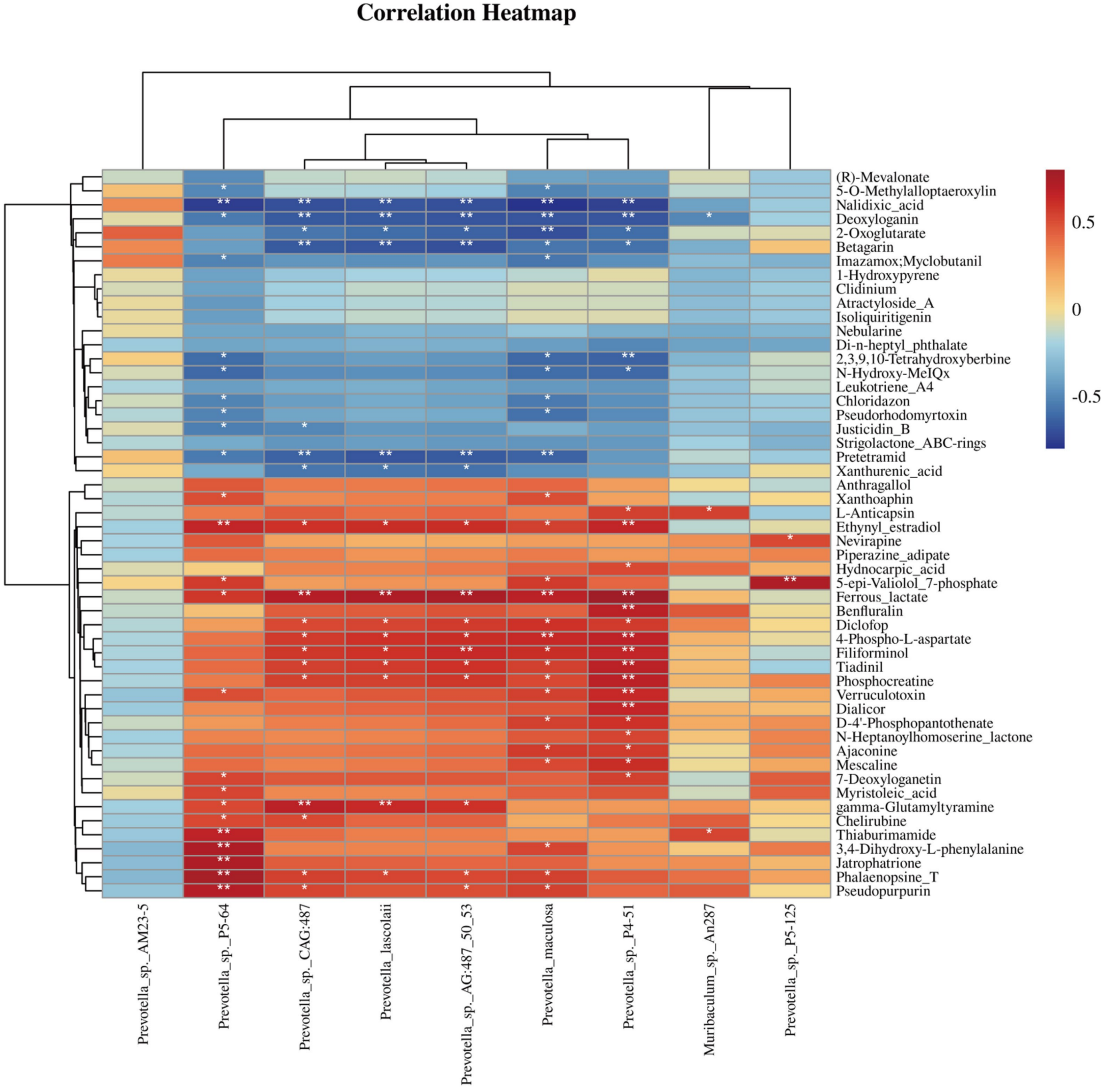
Heatmap of Spearman’s correlation coefficients between the differential abundant gut microbiota and differential metabolites. *p < 0.05, **p < 0.01.

To explore the relationship between cecal microbiota and metabolites, we used Spearman correlation analysis on nine bacterial species with significantly different relative abundances and 51 DMs between CCK and CNT. The correlation matrix evaluation was depicted as a heatmap (Figure 5). We observed a significant positive association of the *Prevotella* sp. P5-64 with cherubins, thiaburimamide, and 3,4-dihydroxy-l-phenylalanine, which are involved in alkaloid synthesis pathways. Moreover, a significant negative association of this microbe was observed with (R)-mevalonate, 5-O-methylalloptaeroxylin, nalidixic acid, and deoxyloganin, which are involved in the terpenoid and steroid biosynthesis pathways. Furthermore, *Prevotella* sp. CAG:487, *P. lascolaii*, *P. maculosa*, and *Prevotella* sp. P4-51 were significantly positively correlated with ferrous lactate (phosphate and phosphate metabolism), phosphocreatine (amino acid biosynthesis), and 4-phospho-p-aspartate (amino acid biosynthesis). These species were negatively correlated with pretermit (tetracycline biosynthesis) and xanthurenic acid (tryptophan metabolism).

### 3.5 TMT-proteomic analysis

**Figure 6:**
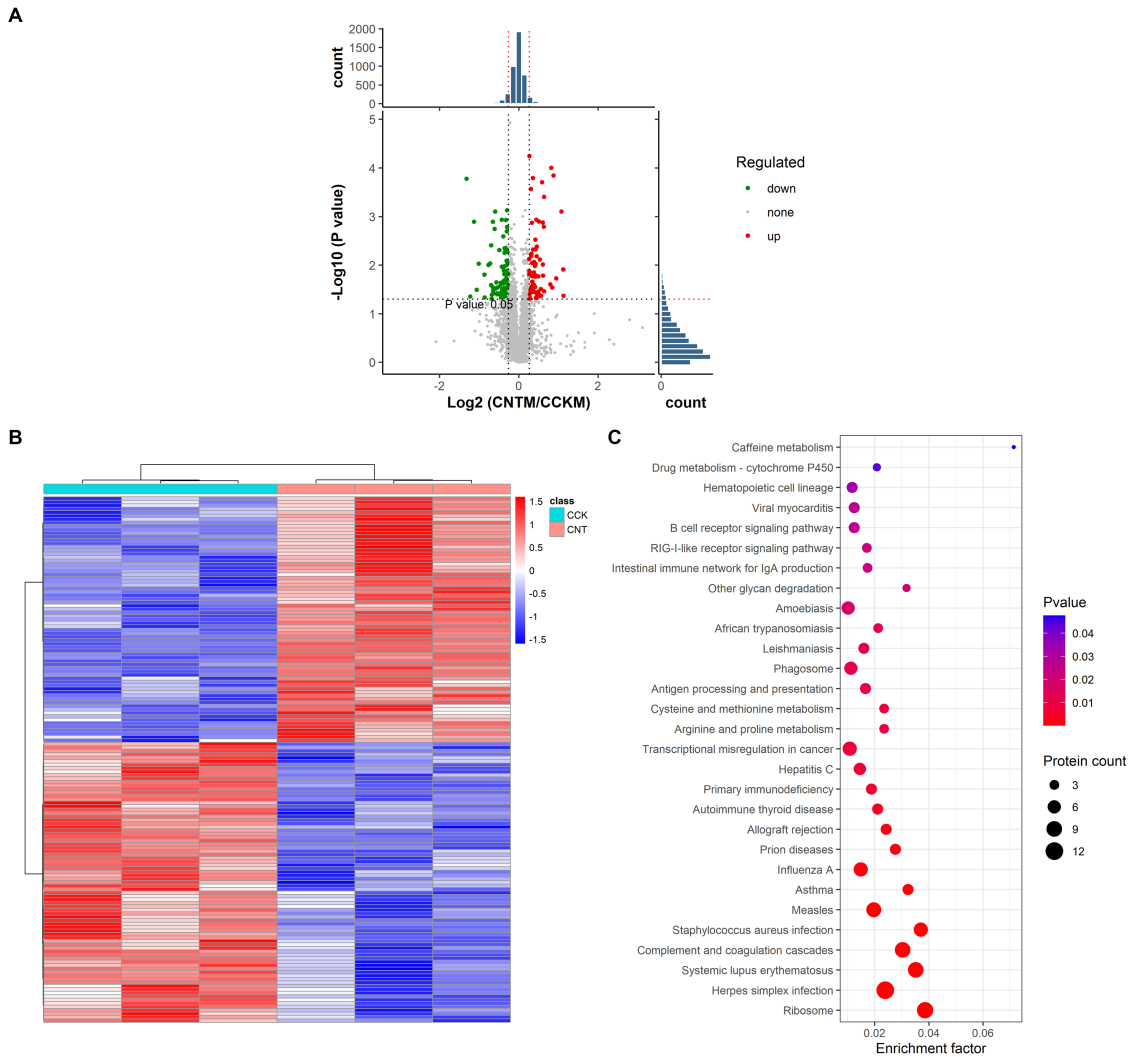
Statistical analysis and functional enrichment of differentially expressed proteins in the bone marrows of CCK and CNT. **(A)** Volcano maps of the differentially expressed proteins. **(B)** Heatmap visualization of the differentially expressed proteins. The color denotes the abundance of proteins, with red and blue colors representing the highest and lowest abundances, respectively. **(C)** Scatter plots representing the KEGG pathway enrichment of the differentially expressed proteins.

We used TMT-labeled quantitative proteomic analysis of chicken bone marrow to elucidate the mechanisms underlying the impact of ANE supplementation on the immunity of Wenchang chickens. Initially, we identified 4,447 highly reliable proteins in bone marrows extracted from CCK and CNT4. These proteins were found to be primarily localized in the cytoplasm, nucleus, and mitochondria (Supplementary Figure S4). Furthermore, using the criteria of fold change >1.2 and *p* < 0.05, we identified 71 upregulated and 81 downregulated proteins between CCK and CNT (Figures 6A,B; Supplementary Table S4). The KEGG enrichment analysis revealed that the differentially expressed proteins primarily enriched in the immune network for IgA production and B cell receptor signaling pathway (Figure 6C).

## 4 Discussion

Areca nut has several pharmacological properties and is used as a traditional medicinal substance. It is known to stimulate digestion, exhibit immunomodulatory effects, and confer infection resistance (10–14). To date, the research on the utilization of areca nut in poultry farming has predominantly focused on the applications of its anti-parasitic properties (23, 24). However, to the best of our knowledge, there is no reported study on the potential beneficial effects of areca nut on the growth, cecal microbiota, and immunity of Wenchang chickens. In the present study, we used ANE as a dietary supplement for Wenchang chickens and investigated its effects in these animals.

First, we observed that ANE treatment significantly enhanced the growth and significantly decreased the F/G ratio in Wenchang chickens. Typically, the growth parameters are not associated with cecal microbiota. However, previous studies showed that supplementing broiler chicken feed with probiotics reduced the F/G ratio and improved the growth of the animals by ameliorating gut barrier function and modulating gut absorption (26). Thus, we investigated whether ANE-induced improvement in the growth of Wenchang chickens is associated with potential alterations in gut microbiota.

Changes in the concentrations of metabolites reflect changes in physiological status and metabolic reactions. In the present study, we found that ANE treatment affected several metabolic pathways and responses in Wenchang chickens, including glycerophospholipid metabolism, D-glutamine and D-glutamate metabolism, HIF-1 signaling pathway, GABAergic synapse, riboflavin metabolism, and the TCA cycle. Among the significantly enriched pathways, we identified several significantly upregulated metabolites in the experimental group, including phospholipids, α-ketoglutarate, lyso PG, irbesartan, ganoderic acid A, and ercalcitriol. These metabolites were primarily associated with gut health. Phospholipids are involved in assembling and maintaning mitochondrial protein complexes and in mitochondrial membrane homeostasis (27). Irbesartan is an angiotensin receptor antagonist that blocks the aldosterone secretion–inducing and vasoconstrictive effects of angiotensin II by selectively blocking angiotensin II receptors, thereby reducing blood pressure and lipid levels (28). Another study showed that irbesartan can regulate oxidative stress and improve antioxidant capacity in rats (29). Ganoderic acids are a class of widely distributed triterpenoids found in *Ganoderma lucidum*. They have been reported to possess antibacterial, antitumor, antioxidant, and antihypertensive properties (30). The upregulation of these metabolites in the ANE-treated Wenchang chickens suggested that the ANE-mediated growth improvement might be associated with an enhanced antioxidative capacity. Similarly, we found that ANE treatment significantly enhanced energy metabolism in Wenchang chickens. This was evident by the modulation of several energy metabolism–related pathways, such as the TCA cycle, pentose and glucuronate interconversions, and lysine biosynthesis. Previous studies have demonstrated that enhancing energy metabolism can improve the poultry growth. For instance, a previous study showed that supplementing the feed with guanidinoacetic acid and betaine can enhance the growth and meat quality of ducks by enhancing energy metabolism (31). The HIF-1 signaling pathway is an important hypoxia-regulated pathway. Under hypoxic conditions, HIF-1 factors are activated, leading to the activation of growth factors that promote energy utilization and metabolism for proliferation and growth (32). For instance, a previous study revealed that the HIF-1 signaling pathway regulates energy metabolism in Tibetan chickens under low-oxygen conditions (33). Our results showed that dietary ANE supplementation significantly activated the HIF-1 signaling pathway, promoting chicken growth even under normoxic conditions. Taken together, ANE treatment improved Wenchang chicken growth by improving lipid metabolism, amino acid metabolism, and energy metabolism.

Gut microbiota plays a pivotal role in enhancing the health and growth of poultry. The diversity in gut microbiota varies by location in the gut, with the highest microbial diversity observed in the cecum. Several studies have reported the beneficial impact of cecal microbiota on the growth performance of chickens (34–37). Our metagenomic sequencing results showed there were no significant differences in cecal microbiota composition at phylum and genus levels between CCK and CNT. This finding suggested that ANE-induced growth improvement in Wenchang chickens might not be mediated via modulation in cecal microbiota at the phylum or genus level. However, differential species analysis based on sample abundance revealed an upregulation of *Prevotella lascolaii*, *Prevotella maculosa*, *Prevotella* sp. AG:487_50_53, and a downregulation of *Hymenobacter* sp. M3. *Prevotella* is a common bacterial genus found in animal intestines that helps break down proteins and carbohydrates (38). Recent studies have shown that *Prevotella* can also produce succinate, which has been reported to enhance animal immune responses (39). This finding suggested that ANE might elevate the abundance of *Prevotella* spp. in the cecum of Wenchang chickens, enhancing the intestinal capacity for nutrient absorption and digestion and hence improving growth.

To understand the role of gut microbiota in ANE-induced metabolic changes in Wenchang chickens, it is essential to analyze the relationship between metagenomics and metabolomics. Our results revealed that ANE-treated chickens exhibited increased levels of cecum metabolites, including chelerythrine and L-DOPA. Chelerythrine is an alkaloid with antimicrobial, antifungal, and anti-inflammatory properties. It can also inhibit microtubule assembly and interact with DNA (40). L-DOPA is a dopamine precursor and a neuroactivator currently considered one of the most effective therapeutic agents for Parkinson’s disease (41). Studies have shown that L-DOPA enhances neural activity by activating protein phosphatases (42). This was consistent with the traditional brain excitability enhancing and antimicrobial effects of ANE (43).

Furthermore, ANE treatment led to the upregulation of several proteins, such as interferon-induced protein with tetratricopeptide repeats 5 (IFIT5), MHC-BF1, and death domain-associated protein (Daxx), in the bone marrow of Wenchang chicken. IFIT5 is an important member of the interferon-stimulated IFIT gene family. Research has demonstrated that IFIT5 negatively regulates the type I interferon signaling pathway by disrupting the formation of the TBK1-IKKε-IRF3 signaling complex, which plays critical roles in regulating immune responses against pathogens, maintaining homeostasis, and enhancing antiviral innate immune responses (44, 45). MHC-BF1 is a class I loci of the major histocompatibility complex (MHC)-B complex. Previous studies found that BF1 might interact with the killer Ig-like receptors (KIRs) expressed by non-MHC-restricted killer cells and activate natural killer (NK)-like cells, enhancing innate immunity (46). Daxx is a highly expressed multifunctional nuclear protein localized within macrophages. One study indicated that Daxx selectively inhibits interleukin (IL)-6 transcription in lipopolysaccharide (LPS)-induced macrophages via histone deacetylase 1 (HDAC1)-mediated histone deacetylation, thus preventing excessive IL-6 production and modulating inflammatory responses during innate immune processes (47). Thus, our findings indicated that ANE can elevate the expression of immune-related proteins in the bone marrow of Wenchang chickens, thus enhancing their immunity.

## 5 Conclusion

In conclusion, dietary ANE supplementation improved the growth performance of Wenchang chickens. This growth improvement was facilitated by the increase in the relative abundance of *Prevotella* spp. in the cecum of ANE-treated Wenchang chickens. In addition, ANE treatment led to an upregulation in the levels of cecal metabolites, such as phosphocreatine, ferrous lactate, and 4-phospho-L-aspartate, which further contribute to the growth of Wenchang chickens. In addition, ANE also elevated the expression of immunity-related proteins in the bone marrow of Wenchang chickens, such as IFIT5, MHC-BF1, and Daxx. Thus, our results suggested that ANE could be used as a promising feed additive to enhance the growth and health of Wenchang chickens.

## Data availability statement

The datasets presented in this study can be found in online repositories. The names of the repository/repositories and accession number(s) can be found at: https://www.ncbi.nlm.nih.gov/genbank/, PRJNA1000214.

## Ethics statement

The animal study was approved by Experimental Animal Center of Hainan Academy of Agricultural Science. The study was conducted in accordance with the local legislation and institutional requirements.

## Author contributions

SW: Writing – review & editing. HW: Writing – original draft. QJ: Writing – original draft. JD: Writing – review & editing. WD: Writing – review & editing. XK: Writing – review & editing. TX: Writing – original draft. XZ: Writing – review & editing. AF: Writing – review & editing. ZX: Writing – review & editing. YC: Writing – review & editing. ZH: Writing – review & editing. LL: Writing – review & editing. LG: Writing – original draft.
