# Supplementary material for: Supplementation of dietary areca nut extract modulates the growth performance, cecal microbiota composition, and immune function in Wenchang chickens
Source: Front Vet Sci. 2023 Dec 12;10:1278312. doi: 10.3389/fvets.2023.1278312 (PMC10773572; doi:10.3389/fvets.2023.1278312)
Supplement: Supplementary file 1 [file Data_Sheet_1.zip › Figures S1-S4 & Tables S1 and S3.docx]

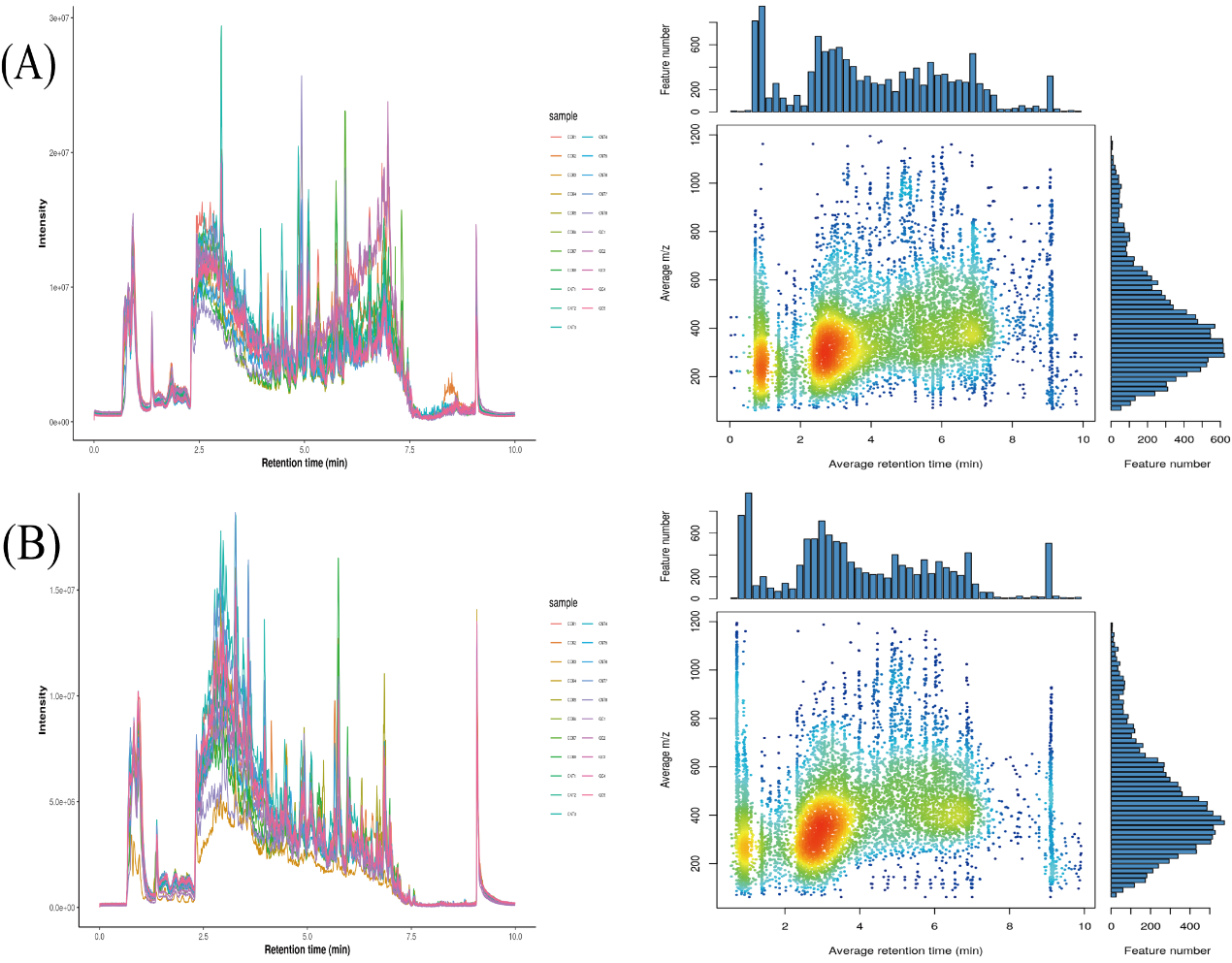


**Supplementary Figure 1 Total Ion Chromatogram and Metabolite quantitative statistics of the detected samples. (a)positive. (b)negitive.**

**Supplementary Table 1 Statistics of differential metabolic ions between compared groups**

| Mode | All | MS2 | HMDB | KEGG | Annotated |
| --- | --- | --- | --- | --- | --- |
| negative | 11881 | 744 | 6470 | 5498 | 7639 |
| positive | 12657 | 468 | 7420 | 6254 | 8339 |

**Supplementary Table 3 The statistical table of metagenomics sequencing**

| Sample Name | Raw Data | Clean Data | GC(%) | Clean(%) |
| --- | --- | --- | --- | --- |
| CCK1 | 32860436 | 26783210 | 48.42 | 81.51 |
| CCK2 | 32856242 | 27357252 | 49.44 | 83.26 |
| CCK3 | 38372360 | 32077122 | 48.32 | 83.59 |
| CCK4 | 36667796 | 30772730 | 50.75 | 83.92 |
| CCK5 | 38318902 | 32322938 | 45.72 | 84.35 |
| CCK6 | 47624774 | 37539588 | 49.06 | 78.82 |
| CCK7 | 49657090 | 40231020 | 48.69 | 81.02 |
| CCK8 | 52558886 | 44561668 | 47.88 | 84.78 |
| CNT1 | 48710926 | 41101262 | 51.36 | 84.38 |
| CNT2 | 52760916 | 42125894 | 46.51 | 79.84 |
| CNT3 | 52578406 | 45330796 | 48.99 | 86.22 |
| CNT4 | 47871374 | 40708052 | 49.61 | 85.04 |
| CNT5 | 61057610 | 51597696 | 48.60 | 84.51 |
| CNT6 | 38901388 | 31911220 | 48.15 | 82.03 |
| CNT7 | 34790028 | 28808000 | 49.34 | 82.81 |
| CNT8 | 36129198 | 29904630 | 48.37 | 82.77 |

**
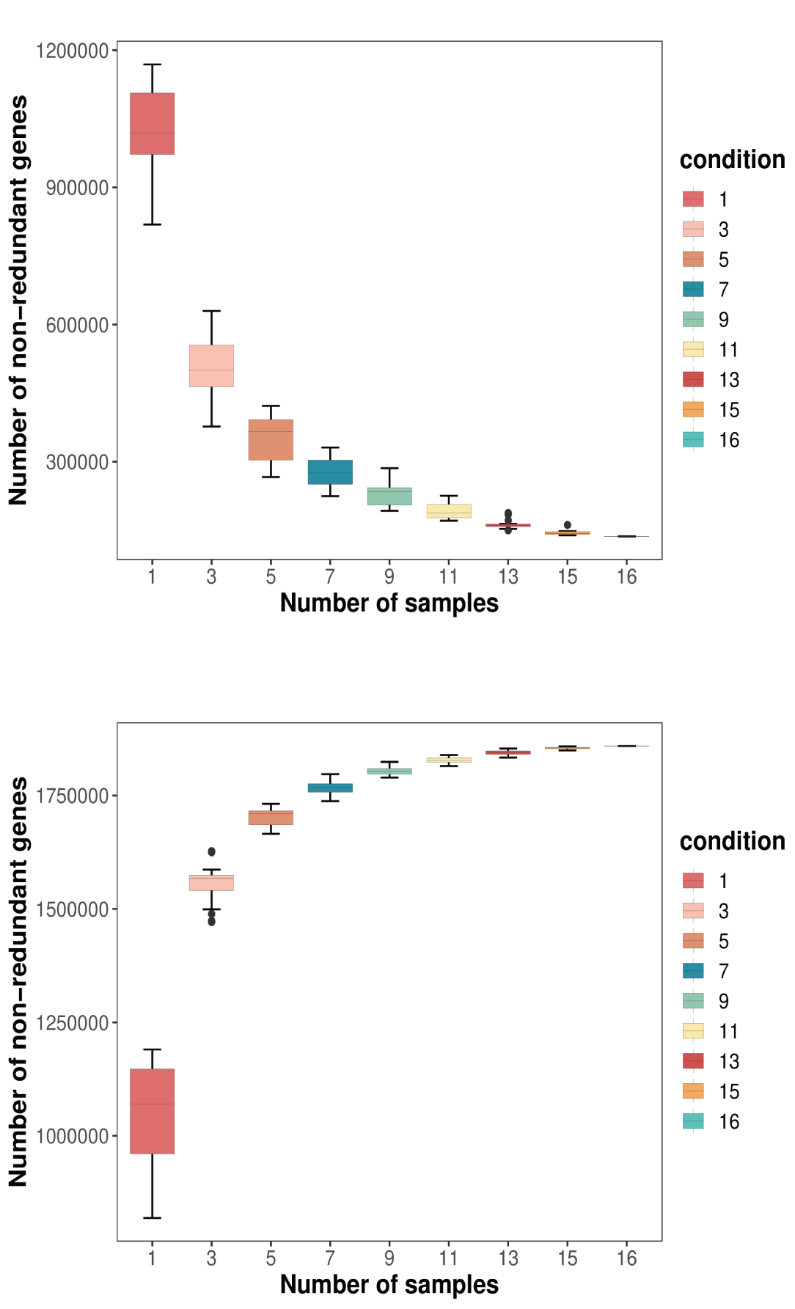
**

**Supplementary Figure 2 Core gene-pan gene dilution curve.**

**
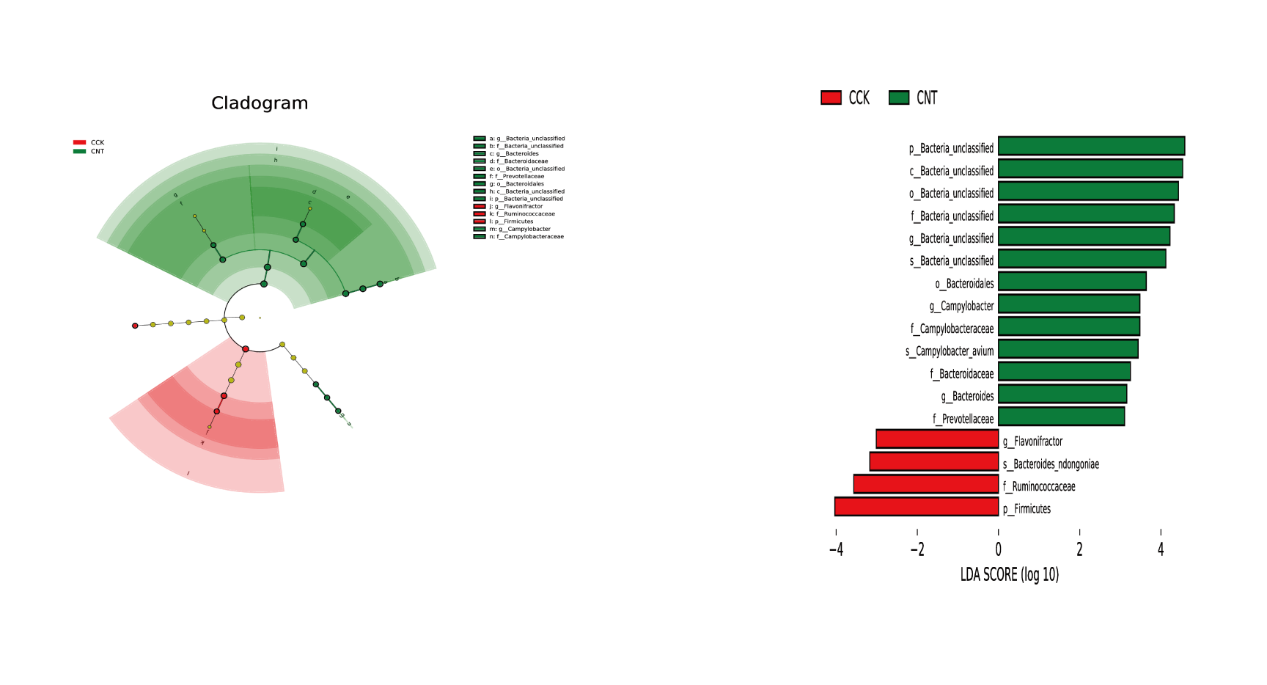
**

**Supplementary Figure 3 Linear effect size (LDA) effect size (LEfSe) analysis at the family level with the threshold set to 3.0.**

**
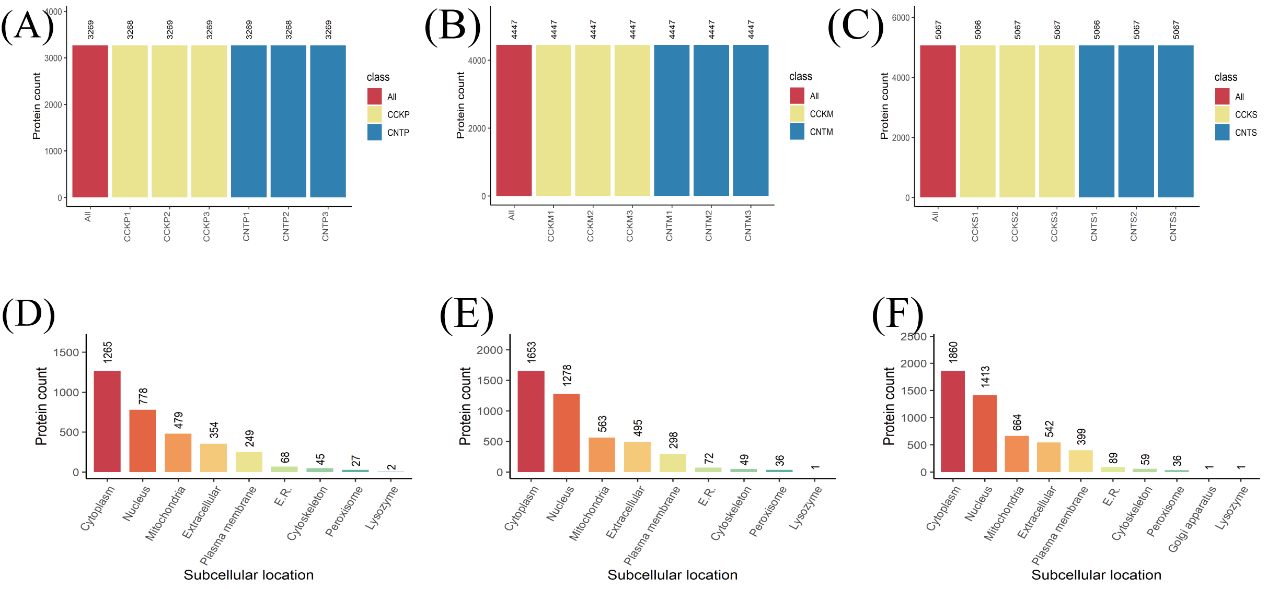
**

**Supplementary Figure 4 Protein number identification and subcellular localization in three tissues. (a-c)** **Protein number in pancreas、bone marrow、lien. (d-f) Subcellular localization in pancreas、bone marrow、lien.**
